# Feather chemicals contain information about the major histocompatibility complex in a highly scented seabird

**DOI:** 10.1098/rspb.2022.0567

**Published:** 2022-05-25

**Authors:** Sarah L. Jennings, Brian A. Hoover, Simon Yung Wa Sin, Susan E. Ebeler

**Affiliations:** ^1^ Graduate Group in Ecology, University of California Davis, Davis CA 95616, USA; ^2^ Department of Viticulture and Enology, University of California Davis, Davis CA 95616, USA; ^3^ Schmid College of Science and Technology, Chapman University, Orange, CA 92886, USA; ^4^ School of Biological Sciences, The University of Hong Kong, Pok Fu Lam Road, Hong Kong SAR

**Keywords:** major histocompatibility complex, feathers, Leach's storm-petrel, chemical communication, mate choice, olfaction

## Abstract

Mate choice informed by the immune genes of the major histocompatibility complex (MHC) may provide fitness benefits including offspring with increased immunocompetence. Olfactory cues are considered the primary mechanism organisms use to evaluate the MHC of potential mates, yet this idea has received limited attention in birds. Motivated by a finding of MHC-dependent mate choice in the Leach's storm-petrel (*Oceanodroma leucorhoa*), we examined whether the chemical profiles of this highly scented seabird contain information about MHC genes. Whereas previous studies in birds examined non-volatile compounds, we used gas chromatography–mass spectrometry to measure the volatile compounds emitted from feathers that potentially serve as olfactory infochemicals about MHC and coupled this with locus-specific genotyping of MHC IIB genes. We found that feather chemicals reflected individual MHC diversity through interactions with sex and breeding status. Furthermore, similarity in MHC genotype was correlated with similarity in chemical profiles within female–female and male–female dyads. We provide the first evidence that volatile chemicals from bird feathers can encode information about the MHC. Our findings suggest that olfaction likely aids MHC-based mate choice in this species and highlight a role for chemicals in mediating genetic mate choice in birds where this mode of communication has been largely overlooked.

## 1. Introduction

The highly polymorphic genes of the major histocompatibility complex (MHC) play a central role in the vertebrate adaptive immune system where they encode for cell surface receptors that bind to and display self- and foreign-derived peptides [1]. MHC alleles in part determine the range of pathogens an individual can respond to, and thus different MHC genotypes are associated with differential survival [2,3] and reproductive success [4–6]. Mating preferences for individuals with high quality or compatible MHC alleles can provide certain fitness advantages including direct benefits like parental care [7] or indirect genetic benefits that enhance the pathogen-resistance of their offspring [8,9]. Furthermore, as close relatives are likely to carry similar genotypes, the MHC may also facilitate inbreeding avoidance [10]. Because genes cannot be directly assessed, MHC-based mate choice requires individuals to detect and evaluate a phenotype that reflects the underlying genotype. Yet, in many species, it remains unclear exactly which phenotypic trait informs MHC-based mate choice.

Owing to its important role in immune function and overall health, MHC can influence a wide range of phenotypes [10,11]. Condition-dependent visual and acoustic traits are often correlated with the MHC [12–16]. However, olfactory cues present in bodily secretions may be more reliable indicators of genotype, because they are directly shaped by the MHC in some species [17,18]. The use of olfaction to evaluate the MHC has been implicated in all major vertebrate groups [19–26], but the vast majority of studies come from laboratory or captive mammals with well-studied olfactory abilities [27].

The avian preen gland and skin produce scented compounds that may serve as a source of olfactory information about the MHC [28]. Birds distribute these compounds throughout their feathers and the resulting cocktail of chemicals can reflect breeding status (e.g. [29]), sex (e.g. [30]) and individual identity (e.g. [31]). However, the idea that birds can sense this chemical information and use it to inform social behaviours has only recently gained traction because birds were widely considered to lack a sense of smell. As the number of species shown to detect and discriminate conspecific odours has grown (reviewed in [32]), a few studies have examined odour-based mechanisms of MHC assessment. Two species, a songbird (song sparrow *Melospiza melodia* [24]) and a seabird (blue petrel *Halobaena caerulea* [23]), can use odour cues to judge MHC similarity. Moreover, the non-volatile chemicals in preen oil contain information about the MHC in both song sparrows [24,33] and another seabird species, black legged-kittiwakes (*Rissa tridactyla* [34]). While these non-volatile chemicals may be precursors to airborne, scented compounds, it is currently unknown whether they can be directly detected by the avian olfactory system. Thus, the search for avian infochemicals should focus on measuring and identifying the volatile chemicals given off by birds.

Our study species, the Leach's storm-petrel (*Oceanodroma leucorhoa*), is particularly well-suited for examining the role of the MHC in avian social signalling. This small, pelagic seabird has strongly scented plumage and an excellent sense of smell [35,36]. Leach's storm-petrels choose their mates based on the MHC class IIB genes [37]. Individuals also possess unique odour profiles, a finding that is consistent with a genetic basis for personal odour [38]. However, we do not yet know whether these individual scents are related to MHC genotype. Here, we tested the hypothesis that information about MHC genotype is reflected in the scent of Leach's storm-petrel plumage. To address this objective, we used locus-specific genotyping of MHC class IIB genes coupled with headspace gas chromatography–mass spectrometry (GC–MS) to measure the chemical profiles of feathers. Unlike previous studies that have focused on non-volatile chemicals, we targeted the volatile compounds associated with the feathers that could potentially be detected via olfaction. We tested the following two predictions: (i) the chemical profiles of individuals contain information about the diversity of their MHC genotypes; (ii) individuals with functionally similar MHC genotypes have similar chemical profiles.

## 2. Methods

### (a) Study site and field methods

We sampled Leach's storm-petrels at a large breeding colony (approx. 39 000 breeding pairs [39]) on Bon Portage Island in Nova Scotia, Canada (43.46° N, −65.75° W). As part of an earlier investigation into MHC-mediated mate choice in this population (2010–2015), blood was collected from a large number of birds and used to determine their MHC genotype and sex (see [37] for detailed methods). To measure chemical profiles, we collected feather samples from 80 incubating adults during the 2016 breeding season. By targeting previously genotyped birds, we were able to sample an equal number of males and females (*n* = 40 per sex) that encompassed most of the common MHC class IIB genotypes in the population (see electronic supplementary material). From each bird, we plucked six small body feathers from approximately 5 cm above the preen gland while wearing clean nitrile gloves. Each sample was placed in a glass vial and kept frozen at −20°C. We transported the feathers on dry ice to the University of California, Davis where they were stored at −80°C prior to analysis.

We checked nests every 3 days to determine the hatch date of each chick. For each adult, we calculated the number of days between the sample date and the hatch date. This value, which we refer to as ‘breeding status’, provided an estimate of how far into the approximately 45-day incubation period each individual was at the time of sampling.

### (b) Chemical analyses

We used previously described methods to measure the chemical profiles associated with Leach's storm-petrel feathers [38]. We analysed samples from each bird in triplicate. Each replicate consisted of two feathers that were weighed and placed into a 10 ml glass vial. Vials were heated to 40°C and we extracted compounds from the headspace of the feathers over 6 h using a 10 mm Twister^®^ stir bar (Gerstel Inc, Germany). We added an internal standard of 0.5 µl of 10 ppm (mg l^−1^) naphthalene-d8 in 100% ethanol to each sample to account for variation in instrument sensitivity across the analysis period. The stir bars were analysed using an Agilent 7890B gas chromatograph (GC) and 5977A mass spectrometer (MS) with a thermal desorption unit (TDU) and cryo-cooled injection system (CIS, Gerstel Inc). The instrument was programmed to optimize peak separation (see electronic supplementary material).

We quantified the peak areas of 80 feather compounds that were previously identified as bird-derived (versus from exogenous sources [38]). We standardized the data from each sample by dividing by the corresponding internal standard peak area and sample mass. We averaged across the three replicate samples to obtain one representative measure per bird (see electronic supplementary material). To prevent the few highly abundant compounds from disproportionately influencing our analysis, we log (*X* + 1)-transformed the data.

The information contained within complex chemical profiles is often encoded by a subset of the compounds present, rather than the entire suite of chemicals [40]. Previous studies have used dimension reduction methods to divide the chemical profile into smaller groups of compounds that can be examined in relation to genetic markers [29,41,42]. This approach offers several advantages: it can allow for the detection of subtly encoded genetic signatures that may be missed in the overall chemical profile, and it can aid in identifying the compounds that are involved in chemical communication. We performed a principal components analysis (PCA) to reduce the chemical profiles of Leach's storm-petrels into several testable variables (*PCA* in R package FactoMineR [43]). A PCA, which uses Euclidean distance, was considered appropriate because the chemical variables were on similar scales after transformation, and they were measured in similar units. To determine the number of principal components (PCs) to retain in our analysis, we compared the results from three statistical approaches (see electronic supplementary material), which indicated that we should proceed with two PCs. From the PCA, we extracted the PC1 and PC2 scores for every individual bird. We also calculated the pairwise difference in PC scores between every dyad of individuals for PC1 and PC2 separately, creating two chemical distance matrices.

### (c) Genetic analyses

We used PCR-based cloning and sequencing to determine the MHC genotype of each bird, focusing on the hypervariable *β* subunit of the MHC class II molecule [37]. Specifically, we targeted the 300 bp gene fragments that span exon 2 in two MHC class II genes, *Ocle-DAB1* and *Ocle-DAB2*, using previously developed locus-specific primers (OcleDAB1Fw 5′-AGAGGGAGGCACAGCAGGAG-3′, OcleDAB2Fw.2­ 5′-GCTGAGAGCACCTTGAGG-3′, OcleDAB12Rv 5′-AGGGAAATGCTCTGCCAAG-3′).

We assessed functional differences between MHC alleles to measure the diversity of each individual's genotype and to quantify MHC distance between individuals. We used five physico-chemical properties to describe the amino acids encoded by the alleles: hydrophobicity (*z*1), steric bulk (*z*2), polarity (*z*3) and electronic effects (*z*4 and *z*5) [44]. Using these properties, we created a matrix of Euclidean distances between amino acids [45,46]. Next, to determine the functional distance between alleles, we calculated the average of the physico-chemical differences across the amino acid sequence for every pair of alleles. The resulting matrix was used to assign MHC diversity and pairwise MHC distance values to the birds.

As a measure of each individual's MHC diversity, we determined the distance between the alleles that comprise their genotype, with higher values reflecting larger functional differences between the alleles and thus a more diverse genotype. We also constructed matrices based on the maximum distance between the genotypes of every dyad of individuals. This provided a measure of pairwise MHC distance between individuals, with lower values indicating dyads with more similar MHC genotypes (see electronic supplementary material). We determined the values for both individual MHC diversity and pairwise MHC distance in three different ways: at each MHC IIB locus separately—*Ocle-DAB1* and *Ocle-DAB2*—and when considering both loci together. We used a locus-specific approach because our previous mate choice analysis had highlighted an important role for the *Ocle-DAB2* in mate choice decisions [37]. However, the mechanisms by which MHC affects odour profiles are likely influenced by multiple MHC genes, and there is evidence to suggest both IIB loci are translated into proteins in this species [47], so we also calculated the genetic measures considering both loci.

We also measured genome-wide heterozygosity at 2514 loci using restriction site-associated DNA sequencing for 312 adults [48]. We examined the relationship between MHC heterozygosity and genome-wide heterozygosity to determine whether genome-wide variation may explain the patterns between MHC and the chemical profiles.

### (d) Statistical analyses

We used linear models to determine whether the chemical profiles of individuals reflect the diversity of their MHC genotype. In total, we examined six models that included either the PC1 or PC2 chemical scores of individuals as the response variable and had one of the three MHC diversity measures as an explanatory variable: diversity at *Ocle-DAB1*, *Ocle-DAB2* and at both MHC IIB loci. Other explanatory variables included in all models were sex, breeding status and the two-way interactions between sex and the measure of MHC diversity, and breeding status and the measure of MHC diversity.

We assessed whether pairwise MHC distance at *Ocle-DAB1*, *Ocle-DAB2* and both MHC IIB loci is correlated with distance in chemical profiles as described by pairwise differences for PC1 and PC2. Specifically, we looked for positive covariance between the genetic and chemical distance matrices to indicate that individuals with similar MHC genotypes have similar chemical profiles. We implemented partial Mantel tests, which allowed us to test the significance of each PC while controlling for the influence of the other, and generated *p*-values using 10 000 randomizations of the data (*mantel* in R package ecodist [49]). Gene–odour covariance may be limited to one sex (e.g. [41]), so we performed separate tests using male–male (M–M) dyads and female–female (F–F) dyads to test for relationships within males and within females, respectively. A Mantel test was not possible on the matrix of male–female dyads (M-F), which was not square, so we used a Spearman's partial correlation test with 10 000 permutations (*pcor.test* in R package RVAideMemoire [50]). A similar approach has been used to analyse mixed-sex dyads in comparable studies [26,33,34]. The pairwise difference in breeding status between individuals was included as a covariate matrix in all the models.

For the Mantel tests where we found a significant positive correlation, we used the BIO-ENV procedure (*bioenv* in R package vegan [51]) to identify the specific compounds that maximized the relationship between the MHC and chemical distance [52]. The user can specify a maximum number of variables to consider; we tested groups of up to six compounds. This process offers an alternative approach to a PCA for determining which compounds in the chemical profile are potentially responsible for signalling MHC genotype.

All statistical analyses were performed using R v. 4.1.2 [53]. We assessed significance using two-tailed tests. For the linear models and Mantel tests, we applied Bonferroni corrections to account for multiple comparisons, so only very strong relationships remained significant (linear models: adjusted *p* = 0.05/6 = 0.008; Mantel tests: adjusted *p* = 0.05/9 = 0.0056).

## 3. Results

### (a) Chemical profiles and MHC genotypes

The first two PCs cumulatively explained 67% of the variation in the chemicals associated with Leach's storm-petrel feathers (electronic supplementary material, figure S3). PC1 was correlated with several long chain esters (electronic supplementary material, table S1). Nine compounds were strongly correlated with PC2, including the fatty alcohol 2-ethyl 1-hexanol, four even-chained fatty acid ethyl esters (C12, C14, C16 and C18) and four unidentified compounds that contained *m/z* 88 and 115 as the most abundant ions in their mass spectra (electronic supplementary material, table S1).

The PC1 and PC2 scores of males and females did not differ (PC1: two sample *t*-test: *t*_78_ = 1.920, *p* = 0.059; PC2: two sample *t*-test: *t*_78_ = 0.067, *p* = 0.957). Individuals on average possessed 3.16 ± 0.79 (mean ± s.d.) unique MHC alleles (range: 2–4 alleles). MHC diversity at the *Ocle-DAB1* locus was not linked with diversity at the *Ocle-DAB2* locus (*r* = 0.150, *p* = 0.182). In total, the 80 birds in our dataset represented 55 unique MHC IIB genotypes. The functional diversity of MHC genotypes did not differ between males and females (two sample *t*-test: *t*_78_ = 1.635, *p* = 0.106). The correlation between MHC heterozygosity and genome-wide heterozygosity was low for both IIB loci (*Ocle-DAB1*: *r* = 0.087; *Ocle-DAB2*: *r* = 0.120), so the MHC is unlikely to be an indicator of the background genetic diversity.

### (b) Chemical profiles and individual MHC diversity

**Figure 1.**
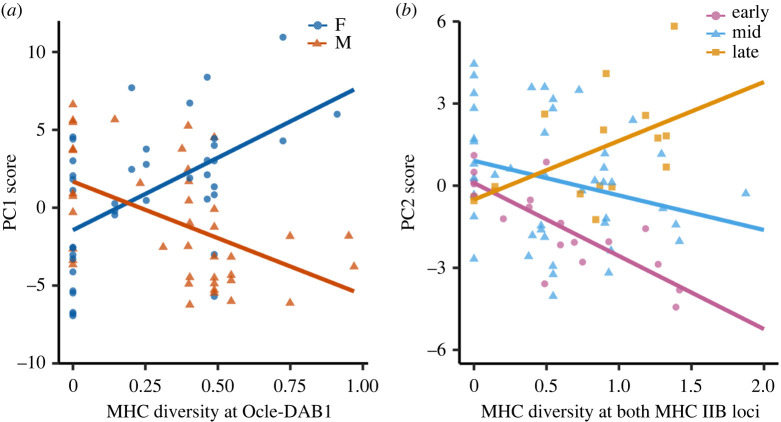
Linear relationship between chemical PC scores and MHC diversity. (*a*) Individual PC1 score is explained by diversity at *Ocle-DAB1* in a sex-specific way. (*b*) Individual PC2 score is explained by diversity across both MHC IIB loci through an interaction with breeding status. Breeding status is represented by three categories with ‘mid' showing individuals in the mean breeding stage, ‘late’ showing individuals +1 s.d. above the mean, and ‘early' showing individuals −1 s.d. below the mean. Solid lines show the least-squares regression for each group. Full model outputs provided in table 1. (Online version in colour.)

The PC1 scores of individuals were explained by a significant interaction between MHC diversity at *Ocle-DAB1* and sex (figure 1*a* and table 1; *p* < 0.001). The PC1 scores of males significantly decreased with increasing diversity at the *Ocle-DAB1* locus (slope = −6.96, *p* = 0.002), while females showed the opposite pattern (slope = 9.40, *p* < 0.001). We did not find evidence that the chemicals associated with PC1 reflected diversity at the *Ocle-DAB2* locus or when considering both MHC IIB loci (electronic supplementary material, table S2).

**Table 1.** Linear relationship between individual chemical profiles and MHC diversity. Significant relationships are shown in italics (adjusted *p*-value for significance <0.008).

chemical variable	explanatory variables	estimated coefficient (±s.e.)	95% CI	*p*-value^a^
PC1	*intercept*	−2.474 (1.522)	−5.505, 0.558	
	DAB1	5.833 (3.687)	−1.513, 13.178	0.118
	sex	−1.472 (0.618)	−2.704, −0.240	0.020
	breeding status	0.148 (0.080)	−0.010, 0.307	0.066
	*DAB1 × sex*	8.183 (1.544)	*5.106, 11.260*	*<0.001*
	DAB1 × breeding status	−0.278 (0.204)	−0.684, 0128	0.177
adjusted *R*^2^ = 0. 292, *F* = 7.506 (d.f.. = 5, 74), *p* < 0.001				
PC2	*intercept*	0.324 (0.974)	−1.617, 2.266	
	both Loci	2.243 (1.171)	−0.091, 4.578	0.152
	sex	−0.162 (0.360)	−0.879, 0.555	0.850
	breeding status	0.005 (0.050)	−0.096, 0.105	<0.001
	both loci × sex	0.204 (0.462)	−0.717, 1.125	0.577
	*both loci* × *breeding status*	−0.181 (0.062)	*−0.304, −0.058*	*0.004*
adjusted *R*^2^ = 0. 227, *F* = 5.651 (d.f = 5, 74), *p* < 0.001				

^a^*p*-values for explanatory variables obtained using an ANOVA with Type III Sum of Squares.

Individual chemical variation at PC2 was explained by a significant interaction between diversity across both MHC IIB loci and breeding status (figure 1*b* and table 1; *p* = 0.004). To assist with the interpretation of this interaction effect, we plotted breeding status as a categorical variable with the mean breeding status (mid-incubation), +1 s.d. above the mean (late incubation), and −1 s.d. below the mean (‘early incubation', figure 1*b*). Birds in early- and mid-incubation have PC2 scores that decrease with increasing diversity across both MHC IIB loci. Late-incubation birds show the opposite relationship; their PC2 scores increase with increasing genetic diversity across both loci. When considering each MHC locus separately, the chemicals associated with PC2 were also related to genetic diversity through an interaction with breeding status, but this relationship was not significant after applying corrected *p*-values (electronic supplementary material, table S2).

### (c) Relationships between chemical and MHC distance

**Figure 2.**
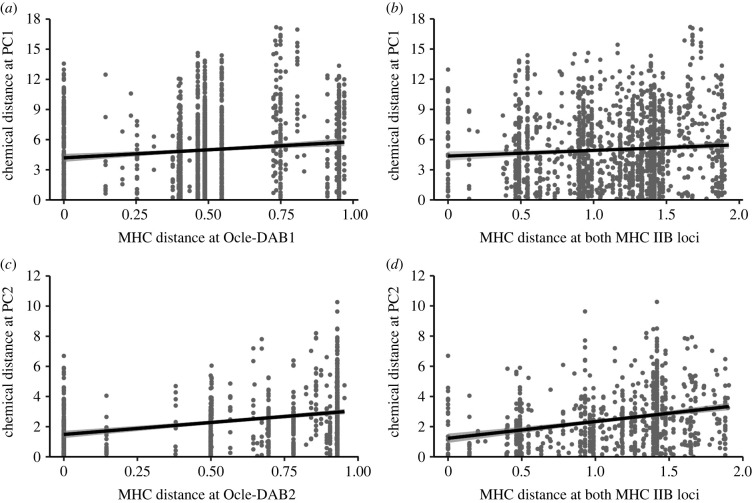
Relationships between pairwise MHC distance and pairwise chemical distance in dyads of Leach's storm-petrels. In M –F dyads there is a significant relationship between pairwise chemical differences in PC1 scores and MHC distance at (*a*) *Ocle-DAB1* and (*b*) both MHC IIB loci. In F –F dyads, there is a significant positive correlation between pairwise chemical differences in PC2 scores and MHC distance at (*c*) *Ocle-DAB2* and (*d*) both MHC IIB loci. Solid lines show the least-squares regression with 95% confidence interval. Full model outputs provided in table 2.

Chemical similarity covaried with MHC similarity in both M–F and F–F dyads (figure 2 and table 2). We found that chemical distance at PC1 was positively correlated with genetic distance at *Ocle-DAB1* (*ρ* = 0.145, *p* < 0.001, figure 2*a* and table 2) and across both MHC IIB loci in male–female dyads (*ρ* = 0.083, *p* = 0.002, figure 2*b* and table 2). There were no relationships between chemical distance at PC2 and any of the genetic distance matrices in mixed-sex dyads.

**Table 2.** Partial Mantel tests show the relationship between chemical distance (PC1 or PC2) and genetic distance (*Ocle-DAB1*, *Ocle-DAB2* or both IIB loci) in M–M and F–F dyads. Spearman partial correlation permutation tests show the relationship between chemical and genetic distance in M–F dyads. Significant positive correlations are shown in italics (adjusted *p*-value for significance <0.0056). Correlation coefficient for F–F and M–M dyads is Mantel *r*, for M–F dyads it is Spearman's rho.

group of dyads	genetic distance	no. dyads	test	chemical distance PC1		chemical distance PC2	
				correlatio*n* coefficient	*p-*value	correlation coefficient	*p-*value
M–M	*Ocle-DAB1*	435	partial Mantel	−0.051	0.396	−0.054	0.457
	*Ocle-DAB2*	435	partial Mantel	−0.014	0.798	−0.162	0.021
	both IIB loci	435	partial Mantel	−0.056	0.338	−0.146	0.040
F–F	*Ocle-DAB1*	435	partial Mantel	0.047	0.509	0.173	0.065
	*Ocle-DAB2*	435	partial Mantel	−0.007	0.926	0.349	*<0*.*001*
	both IIB loci	435	partial Mantel	0.046	0.555	0.296	*0*.*001*
M–F	*Ocle-DAB1*	900	partial Spearman	0.145	*<0.001*	0.018	0.566
	*Ocle-DAB2*	900	partial Spearman	0.011	0.782	0.004	0.982
	both IIB loci	900	partial Spearman	0.083	*0*.*002*	0.005	0.947

Within females (F–F dyads), chemical distance at PC2 positively covaried with MHC distance at the *Ocle-DAB2* locus (*r* = 0.296, *p* = 0.001, figure 2*c* and table 2) and when considering both MHC IIB loci (*r* = 0.349, *p* < 0.001, figure 2*d* and table 2). Chemical distance at PC1, however, had no relationship with genetic distance in F–F dyads (table 2). In males (M–M dyads), we found no evidence of positive covariation between chemical and genetic distance matrices (table 2).

The BIO-ENV process identified the chemicals that maximized the gene–odour covariance at *Ocle-DAB2* and across both MHC loci in females. The best models for both genetic measures used 6 compounds and resulted in slightly higher correlations than the original models that used the chemicals represented by PC2 (table 3; see electronic supplementary material, table S3 for full results). The BIO-ENV process selected several compounds that were highly correlated with PC2, but it also highlighted a possible role for an alkane (heptadecane), three ketones (acetophenone, 2-octanone and 6-methyl-5-hepten-2-one) and a benzene derivative (styrene) that were not strongly associated with either of our retained PCs.

**Table 3.** The top models from the BIO-ENV procedure that identified the subset of chemicals that maximized the correlation between chemical and genetic distance matrices in F –F dyads for genetic distance at *Ocle-DAB2* and at both MHC IIB loci. Compound names in italics were strongly correlated with PC2.

group of dyads	genetic distance	Mantel *r*	no. compounds	compound names
F–F	*Ocle-DAB2*	0.438	6	styrene, 6-methyl-5-hepten-2-one, acetophenone, *unidentified 5,* heptadecane, *ethyl tetradecanoate*
F–F	both MHC Loci	0.416	6	styrene, 2-octanone, *2-ethyl-1-hexanol*, *ethyl decanoate, unidentified 5,* heptadecane

## 4. Discussion

We found support for our hypothesis that the chemical profiles of Leach's storm-petrels contain information about MHC genotype. Our analyses revealed that the feather-associated chemicals reflect individual MHC diversity in a sex-specific- and breeding-status-dependent manner. We also found that similarity in chemical profiles was correlated with MHC similarity in F–F and M–F dyads. These findings are consistent with olfaction as a mechanism for MHC-dependent mate choice in this species. While MHC-associated chemosignals have previously been identified in the non-volatile components of avian preen oil [24,33,34], we present the first evidence showing that the volatile feather compounds suitable for detection by the avian olfactory system also reflect MHC genotype.

Our study was in part motivated by a finding that male Leach's storm-petrels make non-random mate choice decisions to breed less frequently than expected with females that are homozygous at the *Ocle-DAB2* locus [37]. This study also found that these less-preferred, homozygous females are associated with lower reproductive success. Males may evaluate female MHC using one of two recognition mechanisms (reviewed in [10]). If they use self-referent matching, their own phenotype would serve as a reference to assess the genotype of a potential mate. In our data, the best support for this mechanism would be a correlation between chemical similarity and MHC similarity at *Ocle-DAB2* in M–F dyads, which would indicate that males could gain information about this locus by comparing the odour of a female with their own scent. While we did not observe this result, we did detect a correlation in M–F dyads across both IIB loci, suggesting that males may be able to use self-referential matching to glean some information about the MHC of females.

Alternatively, males could imprint on a female family member, such as their mother, and reference this template to discriminate potential mates. If imprinting is at play, our finding that female chemical similarity at PC2 covaries with MHC similarity at *Ocle-DAB2* suggests that males may use odours to avoid homozygous females. However, behavioural experiments are needed to thoroughly explore whether male Leach's storm-petrels can use olfaction to discriminate female MHC, and if so, whether they use self-referential and/or imprinting mechanisms. Cross-fostering experiments using nestlings, which readily perform odour preference tests [35], could shed light on olfactory imprinting. Behavioural trials could also help identify which compounds convey information about the MHC. The compounds highlighted here, specifically the fatty acid ethyl esters with high loadings on PC2 and the additional chemicals selected by the BIO-ENV process, are of particular interest in regard to the female MHC signal.

We detected covariance between the MHC and chemical distance in females, but not in males. We also observed a sex-specific relationship between individual MHC diversity at the *Ocle-DAB1* locus and the chemicals associated with PC1. In vertebrates, females are associated with stronger immune responses than males [54,55]. Furthermore, steroid sex hormones have important regulatory effects on the immune system [55,56]. Testosterone can suppress the immune system in males and has been shown to downregulate MHC class II expression [57,58]. In comparison, oestrogen and progesterone may amplify parasite resistance and humoral-mediated immune responses in females, and have been linked with increased expression of MHC class II ([57,59] but see [60]). The individuals in our study were in breeding condition, a phase associated with elevated levels of sex hormones [61]. If female storm-petrels had increased MHC expression compared with males at the time of sampling, the chemical profiles of females may have been more strongly influenced by the MHC, enabling us to detect the signal in one sex but not the other.

In addition to only finding support for gene–odour covariance in certain dyads of individuals, the effect sizes associated with our positive findings were small. Both of these results are consistent with other studies from mammals and birds, which found similar effect sizes and often only detected relationships in certain dyads [26,33,34,62]. The diverse array of factors that affect chemical profiles may explain these findings. In this study, we targeted MHC class IIB, but storm-petrel odour profiles are likely also influenced by other MHC genes (e.g. MHC class IIA or MHC class I). Genome-wide heterozygosity [41,42], as well as interactions between MHC and background genes can also affect odour profiles [63], although there is little evidence of this in our system where the correlation between MHC and genome-wide markers is low. Moreover, avian chemical profiles vary with diet [64] and disease [65]. Thus, there are a multitude of factors that could contribute variability to the data, resulting in low effect sizes. Studies using captive or MHC-congenic species where more of these confounding variables can be controlled may yield stronger results. However, we believe there is significant value in demonstrating support for odour–gene covariance in wild organisms—particularly in a context where birds may be making these discriminations to facilitate mate choice decisions.

Vertebrate chemical profiles change seasonally and may only reflect genetic markers during the breeding season [26,66,67]. The absence of genetic information in chemical profiles during the non-breeding season might be explained by energetic costs associated with producing chemical secretions [67,68], although there is currently limited support for this idea in birds [69]. Our results indicate that MHC diversity is reflected by chemical profiles in a way that changes within the breeding season. Individuals sampled earlier in the incubation period had PC2 scores that decreased with increasing MHC diversity, but as they approached hatching, the relationship appears to switch. This suggests that there are likely complex interactions happening between steroid hormones, the immune system and other aspects of an individual's physiology and behaviour that alter the way chemical profiles reflect genetic markers over time. Samples from courtship and provisioning would be interesting to further explore how the chemical encoding of MHC shifts with changing reproductive state in this species.

The exact mechanisms that caused the observed relationships between preen feather volatiles and the MHC are currently unknown. Both the MHC molecules and the peptides that bind to them can end up in bodily secretions, where they may act as odourants or the precursors of odourants [20,70]. The MHC may also determine an organism's microbiome and indirectly influence the scented compounds produced by the commensal microbiota [71–74]. The microbiome presents a promising avenue for future research in birds because of its emphasis on the volatile compounds that can be detected by the avian olfactory system. Covariation between the MHC and the avian microbiome has been documented in this population of Leach's storm-petrels [75], the blue petrel [76] and the song sparrow [77]. A three-factor analysis incorporating the microbiome, chemical profiles and MHC (e.g. [77]) would be valuable to shed light on the mechanisms at play in the Leach's storm-petrel.

This study adds to a growing body of work demonstrating that odour reflects information on the MHC in wild vertebrates. Our findings highlight chemicals emitted from bird feathers as a potential source of olfactory information that may enable MHC-based mate choice in Leach's storm-petrels. This species exhibits high fidelity to both their mate and nest site. Because they return to the same nest over many years, and individuals also frequently breed next to the same neighbouring birds. Thus, an exciting possibility for future research in this system is the role of MHC odourtypes in facilitating social interactions beyond mate choice, such as the recognition of neighbours and kin. This system has numerous possibilities for further work that could expand our understanding of olfaction as a mechanism for social communication in birds, an area of research still in its infancy.

## Data Availability

All data files and the code to reproduce the analysis are available on the Dryad Digital Repository [78]. Supplementary information is provided in the electronic supplementary material [79].
